# Quantifying MRI frequency shifts due to structures with anisotropic magnetic susceptibility using pyrolytic graphite sheet

**DOI:** 10.1038/s41598-018-24650-2

**Published:** 2018-04-19

**Authors:** Matthew J. Cronin, Richard Bowtell

**Affiliations:** 1 Sir Peter Mansfield Imaging Centre, School of Physics and Astronomy, University Park, Nottingham, NG7 2RD UK; 2 Present Address: Institute of Imaging Science, Vanderbilt University Medical Center, 1161 21st Avenue South, Medical Center North, AA-1105, Nashville, TN 37232-231 USA

## Abstract

Magnetic susceptibility is an important source of contrast in magnetic resonance imaging (MRI), with spatial variations in the susceptibility of tissue affecting both the magnitude and phase of the measured signals. This contrast has generally been interpreted by assuming that tissues have isotropic magnetic susceptibility, but recent work has shown that the anisotropic magnetic susceptibility of ordered biological tissues, such as myelinated nerves and cardiac muscle fibers, gives rise to unexpected image contrast. This behavior occurs because the pattern of field variation generated by microstructural elements formed from material of anisotropic susceptibility can be very different from that predicted by modelling the effects in terms of isotropic susceptibility. In MR images of tissue, such elements are manifested at a sub-voxel length-scale, so the patterns of field variation that they generate cannot be directly visualized. Here, we used pyrolytic graphite sheet which has a large magnetic susceptibility anisotropy to form structures of known geometry with sizes large enough that the pattern of field variation could be mapped directly using MRI. This allowed direct validation of theoretical expressions describing the pattern of field variation from anisotropic structures with biologically relevant shapes (slabs, spherical shells and cylindrical shells).

## Introduction

Magnetic susceptibility is an important source of contrast in magnetic resonance imaging (MRI), with local spatial variations in the susceptibility of tissue affecting both the magnitude and phase of the measured signals^1,2^. This contrast, which results from the spatially varying magnetic fields produced by the weak tissue magnetization that is induced by the strong, spatially uniform magnetic field of an MRI scanner, has generally been interpreted by assuming that tissues have an isotropic (i.e. scalar) magnetic susceptibility. Recent work has however shown that the anisotropic magnetic susceptibility of some ordered biological tissues, such as myelinated nerves and cardiac muscle fibers, gives rise to unexpected image contrast^3–9^. This behavior occurs because the spatial patterns of field variation generated by microstructural elements formed from material of anisotropic magnetic susceptibility (in which susceptibility is described by a 2^nd^ order tensor) are quite different from those predicted by modelling the effects in terms of isotropic magnetic susceptibility^6,7,9–11^.

The effects of susceptibility anisotropy underlie the orientation dependent T_2_*-decay rate and the non-linear phase evolution of the signal seen in white matter of the brain^12–14^, as well as the orientation- and echo-time- dependent variation of the apparent magnetic susceptibility of some tissues that has been measured in quantitative susceptibility mapping (QSM) experiments^3,5,8,15–17^. In particular, orientation dependent magnetic susceptibility has been identified in white matter of the brain^5^ and cartilage of the knee^15^ in *in vivo* experiments on human subjects and in multiple other tissues, including cardiac muscle^3^ and kidney^8^, in experiments on post mortem tissue, and the technique of susceptibility tensor imaging^9,18^ is based on relating the field variation measured with the object arranged at multiple angles to the applied field, to a voxel-level model of the anisotropic magnetic susceptibility.

Measurements of the evolution of the phase and magnitude of the signal in the presence of microstructural features formed from material of anisotropic magnetic susceptibility potentially provide a new method for probing tissue microstructure^6,7,19–22^. In MR images of tissue, such features are generally manifested at a microscopic, sub-voxel length-scale, so the patterns of field variation that they generate cannot be directly visualized. Rather their effects are inferred from the evolution of the signal averaged over multiple microstructural features. Here, we therefore set out to use a material with a strong magnetic susceptibility anisotropy to form structures of known geometry with sizes large enough that the pattern of field variation could be mapped directly using MRI-based field mapping. This allowed direct validation of theoretical expressions describing the pattern of field variation from anisotropic structures of relevant shape^6,7,10^ (slabs, spherical shells and cylindrical shells).

The material which we used for forming the structures was pyrolytic graphite sheet (PGS). This is a novel material composed of pyrolytic graphite embedded in a flexible polymer film (Panasonic Corporation, Kadoma City, Japan) which is designed mainly for use as a heat transfer medium in compact electronic devices. Pyrolytic graphite (PG) is a highly-ordered form of graphite, usually manufactured by chemical vapor deposition, in which the crystallographic c-axis is preferentially oriented perpendicular to the surface of the substrate. As a consequence PG has a highly anisotropic, diamagnetic magnetic susceptibility; the magnitude and degree of anisotropy strongly depend on the manufacturing conditions^23^. In previous MRI-focused work, the magnetic properties of PG have been exploited in manufacturing passive intra-oral diamagnetic shims to reduce signal loss in frontal regions of the brain^24^ and in producing a solid foam with similar diamagnetic susceptibility to that of water^25^, which can be used to reduce susceptibility artefacts in images.

## Theory

PGS is strongly diamagnetic (with magnetic susceptibility *χ*_⊥_) when a magnetic field is applied perpendicular to the sheet, but only weakly diamagnetic (with magnetic susceptibility *χ*_||_), when the field is applied parallel to the sheet^24^. This susceptibility anisotropy can be described by using a cylindrically-symmetric tensor

1 $$\underline{\underline{{\rm{\chi }}}}=[\begin{array}{ccc}{\chi }_{\parallel } & 0 & 0\\ 0 & {\chi }_{\parallel } & 0\\ 0 & 0 & {\chi }_{\perp }\end{array}]=[\begin{array}{ccc}{\chi }_{I} & 0 & 0\\ 0 & {\chi }_{I} & 0\\ 0 & 0 & {\chi }_{I}\end{array}]+[\begin{array}{ccc}-{\chi }_{A}/2 & 0 & 0\\ 0 & -{\chi }_{A}/2 & 0\\ 0 & 0 & {\chi }_{A}\end{array}],$$

where ${\chi }_{I}=\frac{1}{3}({\chi }_{\perp }+2{\chi }_{\parallel })$ and ${\chi }_{A}=\frac{2}{3}({\chi }_{\perp }-{\chi }_{\parallel })$ characterize the isotropic and anisotropic parts of the susceptibility, respectively. If a structure made from PGS is exposed to an applied magnetic field, ***H***, the magnetization, ***M***, is given by ${\boldsymbol{M}}=\,\underline{\underline{{\rm{\chi }}}}{\boldsymbol{H}}$ and the field perturbation, ***H***_***d***_, due to this magnetization can be described as the gradient of a scalar potential: ${{\boldsymbol{H}}}_{{\boldsymbol{d}}}=-\,\nabla {\rm{\Phi }}$, with

2 $$\Phi ({\boldsymbol{r}})=-\,\frac{1}{4{\boldsymbol{\pi }}}{\int }_{{\boldsymbol{V}}}\frac{\nabla ^{\prime} \cdot {\boldsymbol{M}}({\boldsymbol{r}}{\boldsymbol{^{\prime} }})}{|{\boldsymbol{r}}-{\boldsymbol{r}}{\boldsymbol{^{\prime} }}|}{{\boldsymbol{d}}}^{3}{\boldsymbol{r}}{\boldsymbol{^{\prime} }}\,+\frac{1}{4{\boldsymbol{\pi }}}{\oint }_{{\boldsymbol{S}}}\,\frac{{\boldsymbol{M}}({\boldsymbol{r}}{\boldsymbol{^{\prime} }}).d{\boldsymbol{S}}{\boldsymbol{^{\prime} }}}{|{\boldsymbol{r}}-{\boldsymbol{r}}{\boldsymbol{^{\prime} }}|},$$

where the volume integral is taken over the volume of the PGS structure and the surface integral extends over the internal and external surfaces of the structure. If the structure is embedded in a medium then the value of the isotropic susceptibility of the PGS that we use in solving Eq. (2) should be taken relative to that of the medium. We are interested in the NMR frequency perturbation generated when PGS structures are exposed to a spatially uniform magnetic field, ${H}_{0}\hat{{\bf{z}}}$ (where *B*_0_ = *μ*_0_*H*_0_) and since *χ*_||_, *χ*_⊥_ ≪ 1 we can neglect the effect of terms of order *χ*^2^ and above, by setting ${\boldsymbol{M}}=\underline{\underline{{\rm{\chi }}}}{H}_{0}\hat{{\bf{z}}}$ when calculating Φ(***r***), and also only need to consider the component of the field perturbation that is parallel to the applied field (i.e. along z). As a result of the susceptibility anisotropy, the magnitude and direction of the induced magnetization depend on the orientation of the PGS sheet with respect to the uniform field direction. Considering a small volume, Δ*V*, of PGS oriented such that the normal to the sheet lies in the x-z plane and makes an angle Θ to the z-axis, the induced magnetization is given by

3 $${\boldsymbol{M}}={M}_{x}\hat{{\bf{x}}}+{M}_{z}\hat{{\bf{z}}}={H}_{0}(\frac{3{\chi }_{A}}{4}\,\sin \,2{\rm{\Theta }}\hat{{\bf{x}}}+({\chi }_{I}+\frac{{\chi }_{A}}{4}(1+3\,\cos \,2{\rm{\Theta }}))\hat{{\bf{z}}}).$$

This generates a spatially varying perturbation of the z-component of the magnetic flux density outside the PGS which is described by

4 $$\frac{{B}_{dz}({\rm{r}},\theta ,{\rm{\phi }})}{{B}_{0}}=\frac{{\rm{\Delta }}V}{4\pi \,{r}^{3}}(\frac{9{\chi }_{A}}{8}\,\sin \,2{\rm{\Theta }}\,(\sin \,2{\rm{\theta }}\,\cos \,{\rm{\phi }})+({\chi }_{I}+\frac{{\chi }_{A}}{4}(1+3\,\cos \,2{\rm{\Theta }}))(3\,{\cos }^{2}\theta -1)),$$

corresponding to the sum of the *z*-directed magnetic fields from a *z*-oriented magnetic dipole of strength ${m}_{z}=$ ${M}_{z}{\rm{\Delta }}V={H}_{0}{\rm{\Delta }}V({\chi }_{I}+{\chi }_{A}(1-\frac{3}{2}{\sin }^{2}{\rm{\Theta }}))$ and from an *x*-oriented magnetic dipole of strength ${m}_{x}=\,{M}_{x}{\rm{\Delta }}V=$ ${H}_{0}{\rm{\Delta }}V\frac{3{\chi }_{A}}{4}\,\sin \,2{\rm{\Theta }}$, with *r*, *θ* and *ϕ* representing standard spherical polar co-ordinates. Compared with a volume element of material of isotropic susceptibility, the effect of the susceptibility anisotropy is to produce an orientation-dependent modulation of the strength of the dipole field due to magnetization aligned with the applied field and to introduce an additional field perturbation due to a dipole moment that is perpendicular to the field. The amplitude of the perpendicular dipole moment is maximized when the normal to the PGS is oriented at 45° to the field.

The susceptibility anisotropy gives rise to interesting behavior when PGS is shaped to form structures in which the direction of the principal axis of the susceptibility tensor varies in space. In particular, a thin spherical shell of PGS of radius, *R*, and thickness, *t* ≪ *R*, arranged such that the axis of strong diamagnetism is radial, produces a field perturbation of the form

5 $$\frac{{B}_{dz}(r,\theta )}{{B}_{0}}=\{\begin{array}{ll}{\chi }_{I}\frac{{R}^{2}t}{{r}^{3}}(3\,{\cos }^{2}\theta -\,1) & (r > {\rm{a}})\\ {\chi }_{A}\frac{t}{{\rm{R}}} & (r < {\rm{a}})\end{array},$$

where *r* and *θ* in represent spherical polar co-ordinates (see Supplementary Material for derivation of Eqs (5 and 6)).

Similarly, a long cylindrical annulus of radius, *R*, and thickness, *t* ≪ *R* oriented at angle α to the applied *B*_0_-field produces a field perturbation that is described by

6 $$\frac{{B}_{dz}(\rho ,\phi )}{{B}_{0}}=\{\begin{array}{ll}({\chi }_{I}+\frac{{\chi }_{A}}{4}){\sin }^{2}{\rm{\alpha }}\frac{Rt}{{\rho }^{2}}\,\cos \,2\phi  & (\rho  > R)\\ \frac{3}{4}{\chi }_{A}{\sin }^{2}{\rm{\alpha }}\frac{t}{R} & (\rho  < R)\end{array},$$

where *ρ*, *ϕ* and *z*′ represent cylindrical polar co-ordinates defined relative to the cylindrical annulus.

**Figure 1:**
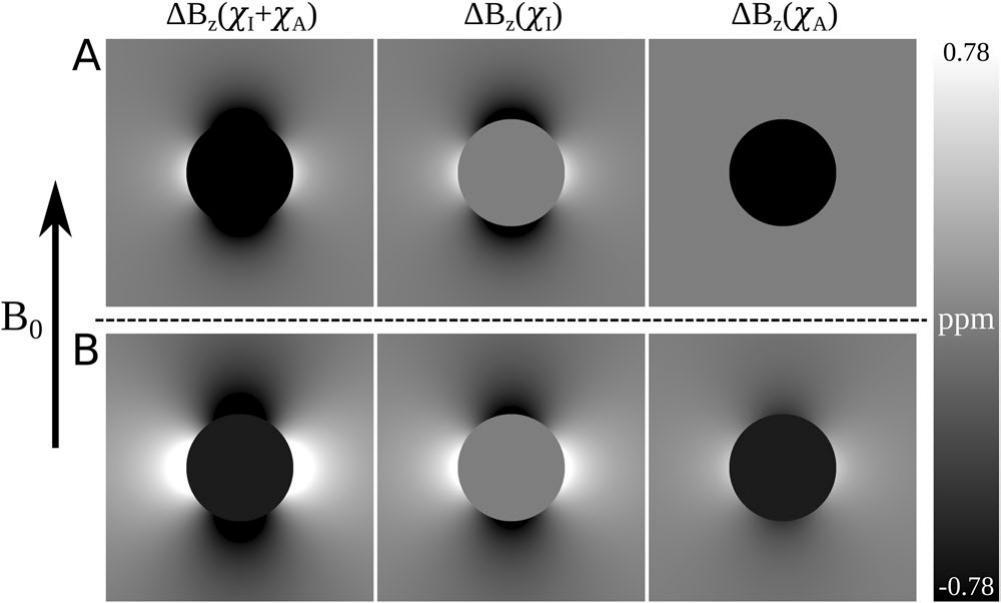
Simulated field maps (Hz) showing the field perturbations due to: (**A**) spherical shells and (**B**) cylindrical shells of pyrolytic graphite sheet exposed to a uniform magnetic field. Individual field maps show the total field perturbation, Δ*B*_*Z*_ (*χ*_*I*_ + *χ*_*A*_), as well as field perturbations due to the isotropic (Δ*B*_*Z*_ (*χ*_*I*_)) and anisotropic (Δ*B*_*Z*_ (*χ*_*A*_)) magnetic susceptibility contributions. For these simulations we used values of *t* = 25 μm, *R* = 10 mm, *χ*_*I*_ = −135 ppm and *χ*_*A*_ = −260 ppm.

Figure 1 shows simulations of the patterns of field variation (in ppm) produced by thin spherical (Fig. 1A) and cylindrical (Fig. 1B) shells of material that were generated using Eqs (5) and (6). The field perturbations due to the isotropic and anisotropic components of the susceptibility are shown separately and then combined for both geometries. For these simulations, we used values of *t* = 25 μm, *R* = 10 mm, *χ*_*I*_ = −135 ppm, and *χ*_*A*_ = −260 ppm, which are representative of the PGS structures used in the experiments reported below, and it is assumed that the cylindrical shell is oriented perpendicular to *B*_0_.

For both spherical and cylindrical shells, the anisotropic component of the magnetic susceptibility generates a uniform field offset inside the structure, whose magnitude depends on the ratio of the shell’s thickness to its radius^7^. The isotropic magnetic susceptibility generates fields of the well-known form outside the shell (dipolar for the spherical shell, and varying as cos 2*ϕ*/*ρ*^2^ for the cylindrical annulus). For the spherical shell, the anisotropic susceptibility does not contribute to the external field perturbation, whilst for the cylindrical shell the anisotropic part of the susceptibility makes a four times smaller contribution to the external field than the isotropic susceptibility. Inspection of Eq. (6) shows that simplistically representing the effect of susceptibility anisotropy by setting *χ*_*A*_ to zero and making the magnitude of *χ*_*I*_ vary with the orientation of the cylindrical shell with respect to the field as sin^2^ α, will produce a spatially varying external field whose amplitude varies as sin^4^ α, with zero field perturbation inside the shell.

## Methods

MRI-based field mapping experiments were carried out at 3T to demonstrate the effects of the anisotropic magnetic susceptibility of shaped structures formed from PGS on the spatial pattern of NMR frequency offsets. Different structures were produced from 25 µm thick PGS (Panasonic EYGS121803). Three- dimensional field maps with 1.5 mm isotropic resolution were acquired using a dual-echo field mapping sequence. For the spherical and cylindrical shell phantoms, the imaging parameters were: TE_1_/TR = 3/40 ms, ΔTE = 1 ms, flip angle = 18°. For the PGS stacks, the imaging parameters were: TE_1_/TR = 3/7.4 ms, ΔTE = 2 ms, flip angle = 8°. All experiments were carried out at room temperature, which was in the range of 18–21 °C.

### Measuring the external field perturbation due to a small PGS stack

To evaluate the field perturbation produced by a small volume of material of anisotropic susceptibility, disks of nominally 25-μm-thick PGS with a diameter of 4.95 mm were stuck together using a small amount of cyanoacrylate glue to form a cylindrical stack of ~5 mm height, using 65.96 ± 0.03 mg of pre-cut disks with a mean mass of 0.984 ± 0.006 mg per disk. The mean thickness of the PGS disks was measured to be 29 ± 0.5 μm and the volume, Δ*V*, of PGS in the stack was calculated to be (3.74 ± 0.22) × 10^−8^ m^3^. The stack was embedded in an agar-filled, perspex sphere which had a diameter of 180 mm. The agar phantom was constructed from 2% (by total weight) agar (Sigma Aldrich) in distilled water and 0.5% NaCl. The values of isotropic magnetic susceptibility which we determined here and in the other experiments described below (which were also made on PGS structures embedded in agar) are therefore measured relative to the susceptibility of agar, which is around −9 ppm. Field maps were generated with the stack oriented at 5 different angles (Θ) to the field. The field variation in a spherical shell centered on the stack with an internal radius of *r*_*i*_ = 10 mm and external radius *r*_*o*_ = 15 mm was analyzed in each of these maps to identify the amplitudes of the field contributions due to *M*_*x*_ and *M*_*z*_. From Eq. (4), the z-component of the field perturbation (in Hz) may be expressed as ${\rm{\Delta }}{B}_{z}={A}_{x}(3/2{r}^{3})(\sin \,2\theta \,\cos \,\phi )+{A}_{z}(1/{r}^{3})(3\,{\cos }^{2}\theta -1)$, where ${A}_{x}={\mu }_{0}\gamma {m}_{x}/4\pi$, and ${A}_{z}={\mu }_{0}\gamma {m}_{z}/4\pi$, with $\gamma =42.576\,{{\rm{MHzT}}}^{-1}$. To find the values of *A*_*x*_ and *A*_*z*_ at each stack orientation, the voxels in the spherical shell were first binned based on their θ values (using 10 bins each of width π/10) and the field at each voxel was multiplied by the value of *r*^3^ to eliminate the dependence on radial co-ordinate. The average value of the scaled field offset (〈Δ*B*_*z*_*r*^3^〉), in each bin was then plotted against (3 cos^2^ *θ* − 1) and linear regression used to estimate the value of *A*_*z*_. Averaging the value of the scaled field multiplied by the value of cos *ϕ* for each voxel in a bin and scaling by the average value of cos^2^ *ϕ*, and fitting the resulting values of $\langle {\rm{\Delta }}{B}_{z}{r}^{3}\,\cos \,\phi \rangle /\langle {\cos }^{2}\phi \rangle$, against 3/2 sin 2*θ* yielded an estimate of *A*_*x*_. Fitting the variation of these amplitudes with stack orientation to 3/2 sin 2Θ or (1 − 3/2 sin^2^ Θ) allowed evaluation of the magnitudes of *χ*_*I*_ and *χ*_*A*_ using Eqs (3) and (4).

### Measuring the field perturbation due to spherical PGS shells

The field perturbation due to spherical shells with anisotropic magnetic susceptibility was assessed by using five thin-walled plastic spheres with radii, *R*, of 5, 10, 12.5, 19 and 25 mm, which were covered with a layer of nominally 25-µm-thick PGS, filled with water, and then set in a 180-mm-diameter spherical agar phantom. Each spherical shell was formed from 10 identical pieces of PGS, with small gaps left between the pieces to reduce the RF shielding of the inside of the sphere by the electrically conducting graphite. A field map of the phantom was generated and the field variation was measured inside each sphere and over a spherical shell outside each sphere with inner/outer radii = 10.5/18, 18/25.5, 22.5/30, 31.5/39 and 40.5/48 mm for the 5, 10, 12.5, 19 and 25 mm radius spheres, respectively. The difference of the mean field over the internal ROI relative to that in the external ROI was calculated for each sphere. A linear fit of these Δ*B*_*z*_ values with respect to 1/*R* was then calculated, reflecting the field variation expected from Eq. (5). In addition, the external field perturbation was analyzed within a spherical shell outside each sphere with inner/outer radii = 7.5/16.5, 12/18, 15/19.5, 24/28.5 and 30/33 mm for the 5, 10, 12.5, 19 and 25 mm radius spheres, respectively. These radii were chosen to avoid artifacts due to small air bubbles trapped on the surface of the shell and to ensure adequate sampling of the field surrounding each sphere. The field map was additionally filtered using SHARP filtering^26^. This technique selectively removes background fields originating from sources outside of a region of interest, which may have a significant effect on these measurements due to the relatively lower amplitude and larger length-scale spatial variation of the external fields. The field at each point within each annulus was multiplied by the cube of the radius (*r*^3^) and then its variation was fitted against 3 cos^2^ *θ* − 1, reflecting the variation expected from Eq. (5). The amplitude of this variation was then used to estimate *χ*_*I*_.

### Measuring the internal and external field perturbations due to cylindrical PGS shells

To measure the field perturbation due to cylindrical shells with anisotropic susceptibility, a phantom was constructed by covering glass tubes with three different external diameters (5, 10 and 15 mm) with a layer of nominally 25-µm-thick PGS, producing hollow cylinder structures. A 7.5 cm length of the tube was covered with PGS, but narrow slots running lengthwise down each cylinder were left uncovered to reduce the RF shielding of the inside of the tubes by the graphite coating. The tubes were filled with agar and embedded in a 180-mm-diameter spherical agar phantom. Field maps were generated with the tubes oriented at angles (α) of 1°, 17°, 33°, 49°, 59°, 73° and 89° with respect to the magnetic field. The field perturbations were measured inside each cylinder and over a cylindrical annulus surrounding each cylinder (length = 15 mm, inner/outer radii ≈7.5/15, 6.0/13.5, and 4.5/12 mm for the 15, 10 and 5 mm diameter cylinders, respectively) at each angle to the field. To minimize errors due to other sources of field variation, mean internal field shifts were calculated relative to the average field in the surrounding cylindrical annulus at each angle and the results were compared to the behavior predicted by Eq. (6). In addition, the external field perturbation was analyzed within a cylindrical annulus of inner radius 7.5 mm and outer radius 15 mm around each tube. The field map was additionally filtered using SHARP filtering^26^. The field at each point within the annulus was scaled by multiplication by the square of the radius (*ρ*^2^) and then its variation was fitted against cos 2*ϕ*, reflecting the variation expected from Eq. (6). This value was then plotted as a function of sin^2^ α, where α is the angle between the axis of the tube and the applied *B*_0_-field.

## Results

### Measuring the external field perturbation due to a small PGS stack

**Figure 2:**
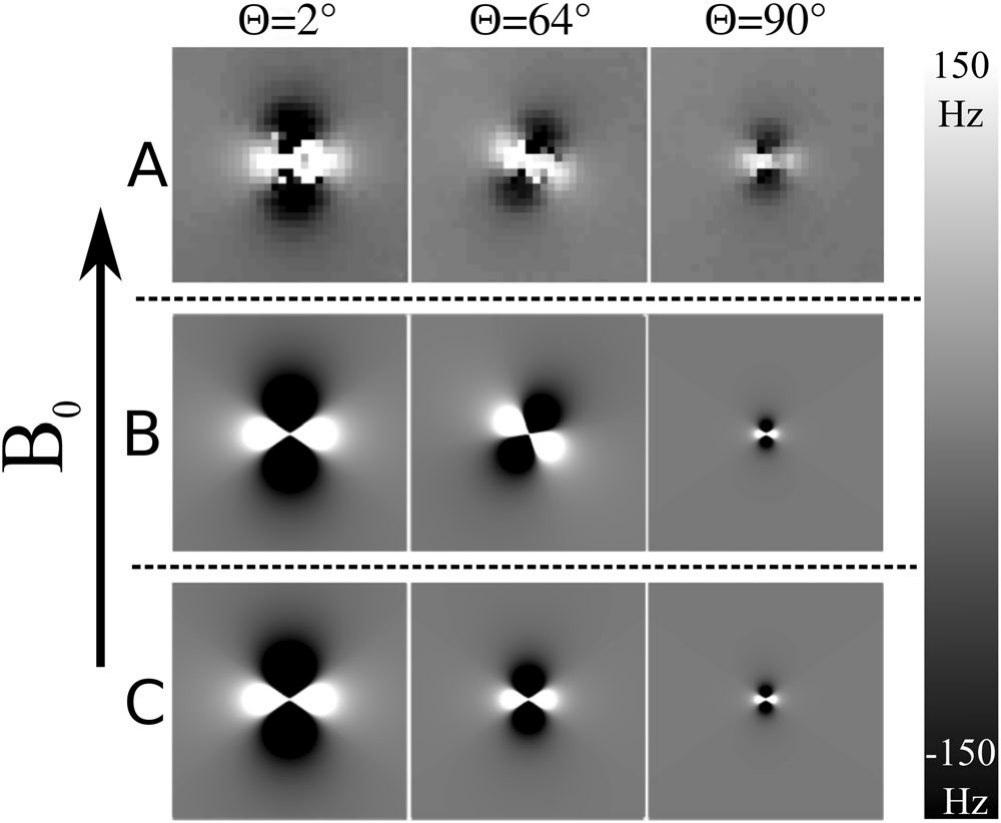
Field maps (Hz) measured at 3T in the x-z plane with the PGS stack at three different orientations (2°, 64°, and 90°) to the *B*_0_-field with the normal to the stack also always lying in the x-z plane. Each image shows a 48 × 48 mm^2^ region cutting through the center of the PGS stack. (**A**) Experimentally measured field maps. (**B**) Simulated field maps taking account of the components of the induced magnetization that are parallel and perpendicular to the applied *B*_0_-field. (**C**) Simulated field maps taking account only of the component of the induced magnetization that is parallel to the applied *B*_0_-field. The simulations were based on values of *χ*_*I*_ = −135 ppm and *χ*_*A*_ = −260 ppm, and a PGS stack volume of 3.29 × 10^−8^ m^3^.

Figure 2A shows field maps measured in the x-z plane with the PGS stack at three different orientations to the ${B}_{0}$-field with the normal to the stack also always lying in the x-z plane. Each image shows a 48 × 48 mm^2^ region cutting through the center of the PGS stack. Simulated field maps are also shown for the cases where the full effect of the anisotropic susceptibility is accounted for (Fig. 2B) and where only the effect of the induced magnetization that is parallel to the magnetic field is considered (Fig. 2C). The simulations used susceptibility values of *χ*_*I*_ = −135 ppm and *χ*_*A*_ = −260 ppm, and a PGS stack volume of 3.29 × 10^−8^ m^3^ and assumed that the induced magnetization was confined to a single voxel. A field perturbation following a standard dipolar field pattern (3 cos^2^ *θ* − 1)/*r*^3^, aligned with *B*_0_, is evident in the experimental measurements made when the normal to the stack is nearly parallel (2°) or perpendicular (90°) to the *B*_0_*-*field, with the amplitude being greater in the parallel case. At the intermediate angle (64°) the field contribution from *M*_*x*_, which varies as sin 2θ cosϕ, is evident from the rotation of the experimentally measured dipolar field pattern. The rotated field pattern is seen in the simulations where the full effect of the anisotropic susceptibility is accounted for (Fig. 2B), but when only the effect of the magnetization that is parallel to the applied field (*M*_*z*_) is considered, the field variation corresponds to a standard dipolar field with an orientation-dependent amplitude (Fig. 2C). The data shown in Fig. 2B was generated using Eq. (4), while the data shown in Fig. 2C were modelled using only the second of the two summed terms in Eq. (4), which represents the contribution of z-directed magnetization only.

**Figure 3:**
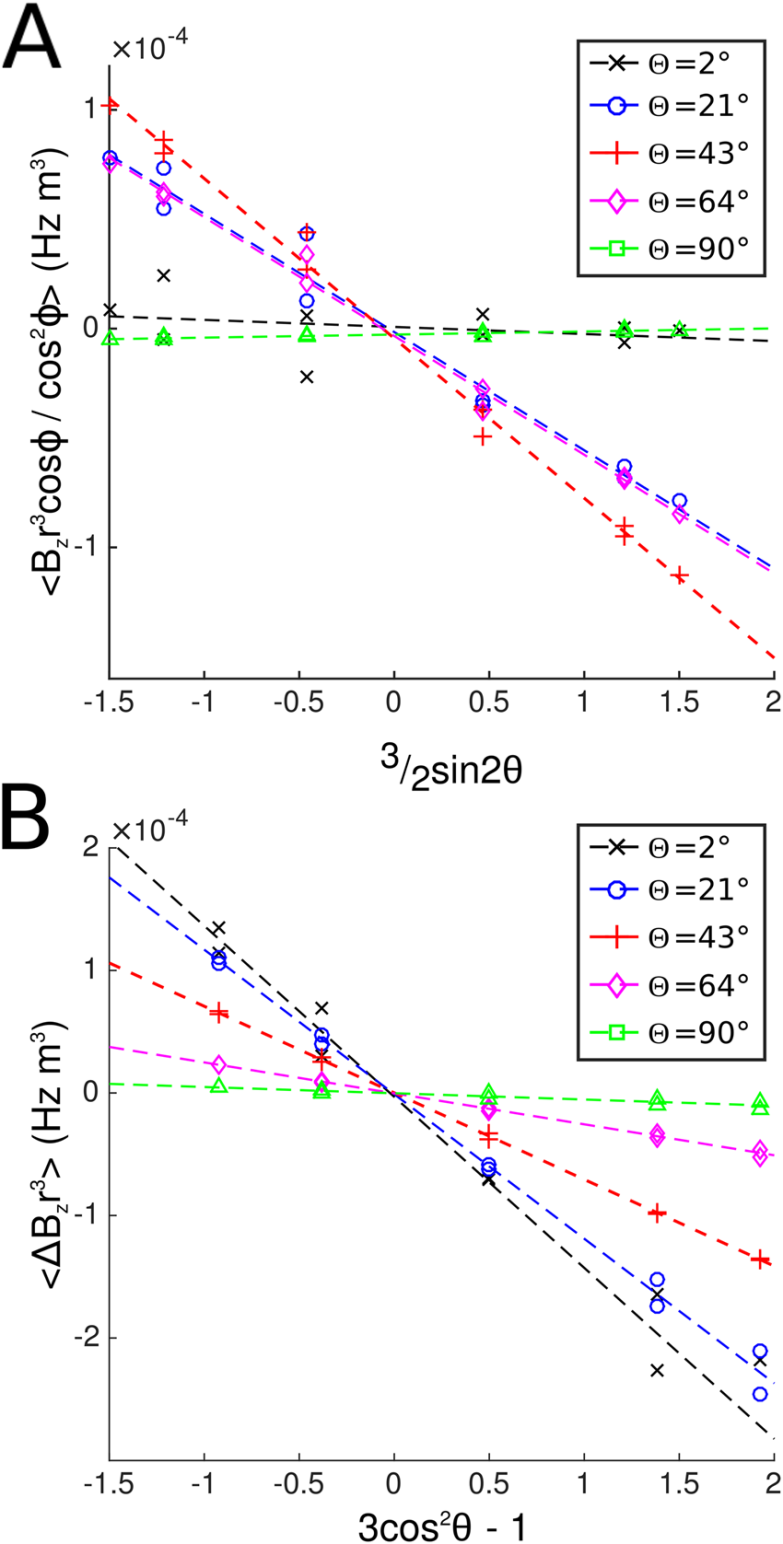
Variation of the field around the PGS slab oriented at different angles (2, 21, 43, 64 and 90°) to the applied field. (**A**) $\langle {\rm{\Delta }}{B}_{z}{r}^{3}\,\cos \,\phi \rangle /\langle {\cos }^{2}\phi \rangle$ with 3/2 sin 2*θ*. (**B**) Variation of 〈Δ*B*_*z*_*r*^3^〉 with (3 cos^2^ *θ* − 1). The slopes of these plots yielded values of *A*_*x*_ and *A*_*z*_, which reflect the strengths of the *x*- and *z*-components of the induced dipole moment.

**Figure 4:**
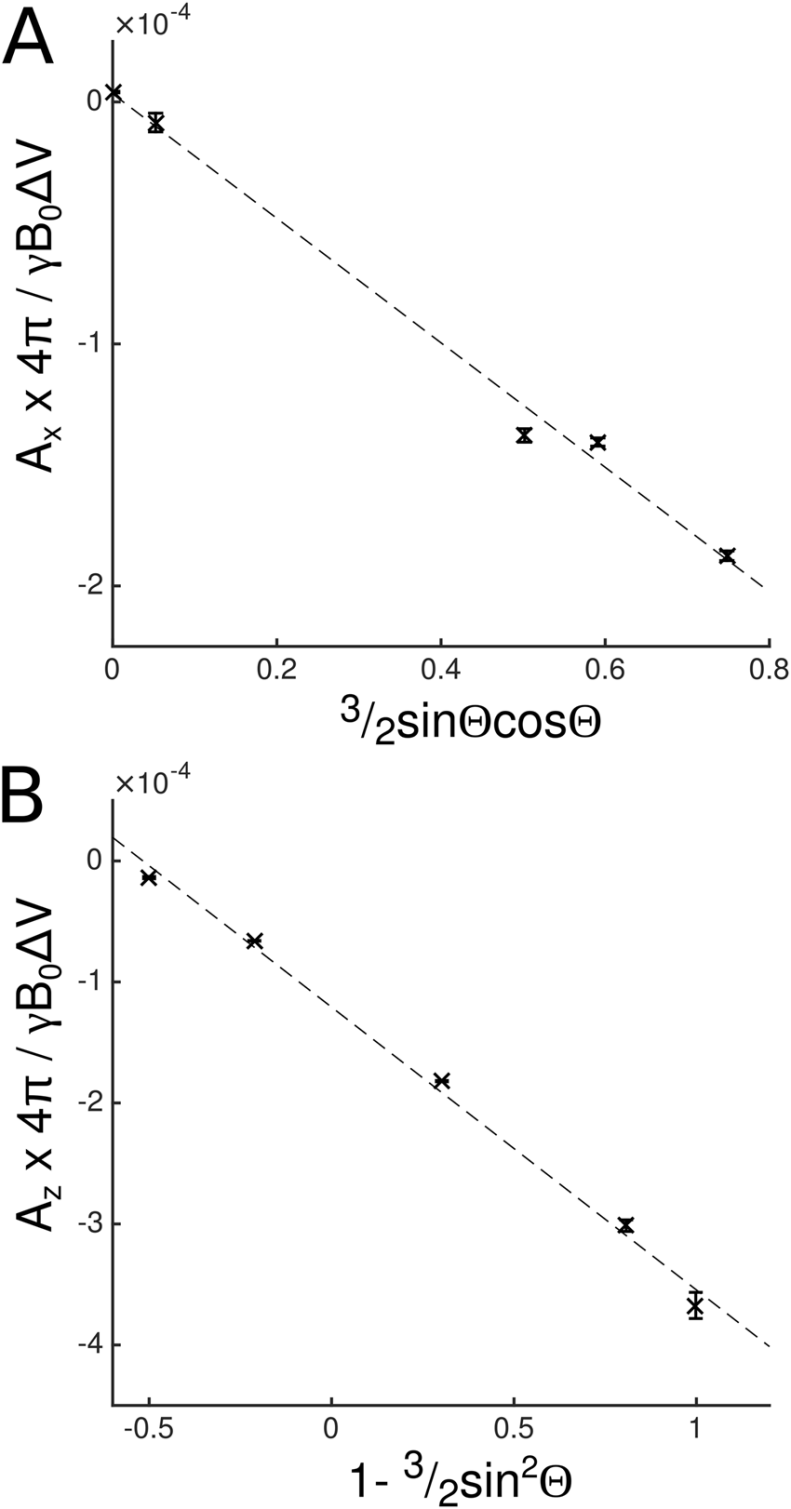
(**A**) Variation of *A*_*x*_ × 4*π*/*γB*_0_Δ*V* with 3/2 sin Θ cos Θ. The slope of this plot is *χ*_*A*_. (**B**) Variation of *A*_*z*_ × 4*π*/*γB*_0_Δ*V* with 1 − 3/2 sin^2^ Θ. The slope of this plot is *χ*_*A*_ and the value when 1 − 3/2 sin^2^ Θ = 0 is *χ*_*I*_. Values of *A*_*x*_ and *A*_*z*_ were derived from Fig. 2 and *γB*_0_Δ*V*/4*π* = 0.38 Hzm^3^.

Figure 3 shows the variation of $\langle {\rm{\Delta }}{B}_{z}{r}^{3}\,\cos \,\phi \rangle /\langle {\cos }^{2}\phi \rangle$ measured in a spherical shell around the PGS stack with 3/2 sin 2*θ* (Fig. 2A), along with the variation of the value of 〈Δ*B*_*z*_*r*^3^〉 with 3 cos^2^ *θ* − 1 (Fig. 3B), for five different orientations of the stack with respect to the *B*_0_-field. The linearity of these plots indicates that the spatially-varying fields produced by the PGS follow the form predicted by Eq. (4). The calculated variation of *A*_*x*_ scaled by *γB*_0_Δ*V*/4*π* = 0.38 Hzm^3^ with 3/2 sin Θ cos Θ is shown in Fig. 4A. The slope of this plot gave a value of *χ*_*A*_ = −257 ± 21 ppm. Figure 4B shows the variation of *A*_*z*_ scaled by *γB*_0_Δ*V*/4*π* with 1 − 3/2 sin^2^ Θ. The slope of this plot gave a value of *χ*_*A*_ = −234 ± 18 ppm, while the intercept gave a value of *χ*_*I*_ = −121 ± 10 ppm.

### Measuring the field perturbation due to spherical PGS shells

**Figure 5:**
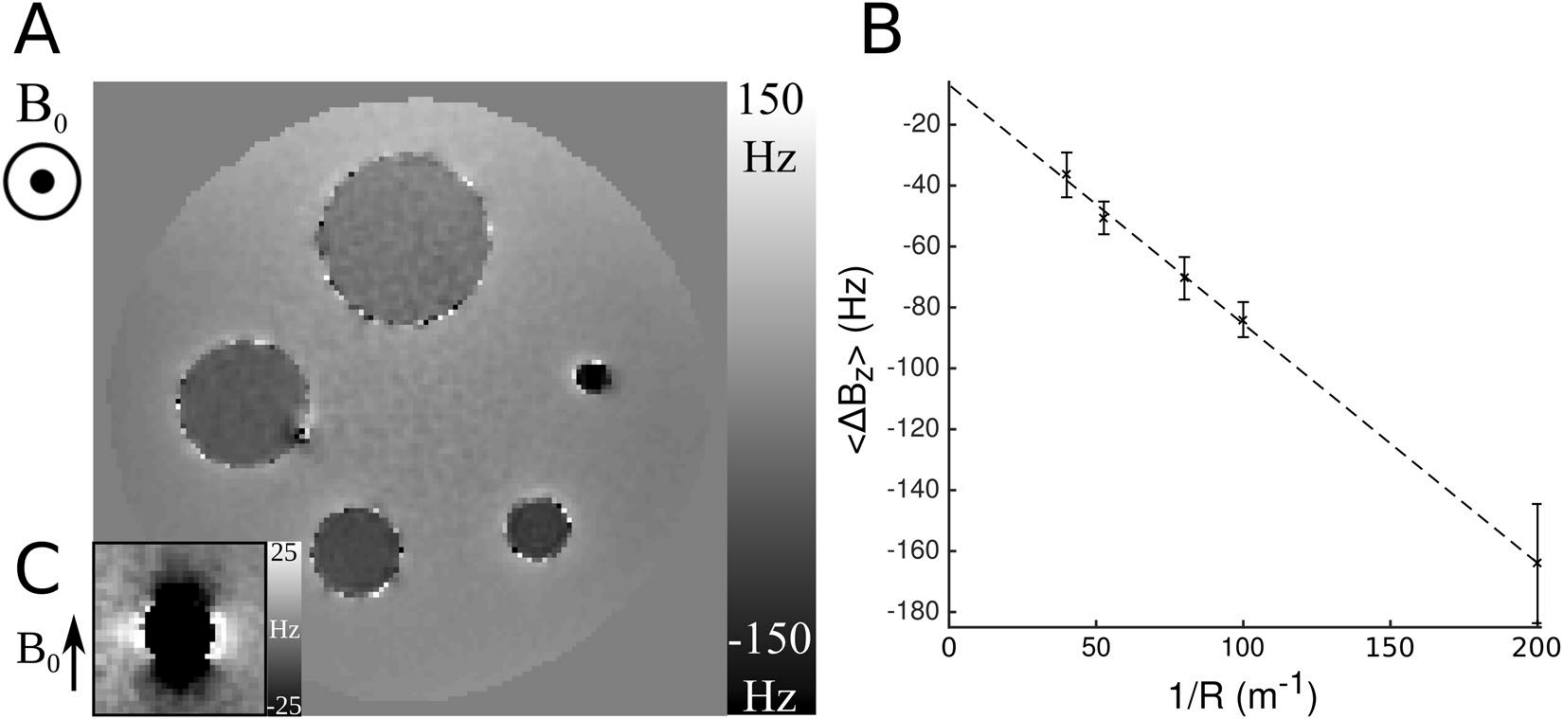
(**A**) Axial cross section through a field map (in Hz) showing the field variation due to five plastic spheres (radii, *R* = 5, 10, 12.5, 19 and 25 mm) coated in nominally 25-μm-thick PGS and embedded in a 180 mm diameter spherical agar phantom. A uniform field offset, which increases with decreasing sphere radius, can be seen inside each shell, with little field perturbation outside each sphere. (**B**) Plot showing the variation of the mean internal field shift due to the spherical shells 〈Δ*B*_*z*_〉 as a function of 1/*R*. Error bars show the standard deviation of the field measured inside each sphere. (**C**) Field variation in field in a ρ-z plane cutting through the 10-mm-diameter sphere. Note that the frequency range shown here is a factor of 6 smaller than that shown in A (with values clipped to ±25 Hz to aid visualization of the external dipolar field pattern).

Figure 5A shows an axial cross-section through the field map spanning the centers of the five spheres. It shows a uniform negative field offset inside each sphere, with a magnitude that decreases with increasing radius, as predicted by Eq. (5). Figure 5B shows the linear variation of the mean internal field shift with 1/*R*. Linear regression yields a fit with R^2^ > 0.99, indicating that inside the shells Δ*B* ∝ *R*^−1^, as predicted by Eq. (5). The slope of the plot is $-\,\gamma {B}_{0}t{\chi }_{A}$, yielding a value of *χ*_*A*_ = −212 ± 16 ppm. Inspection of Fig. 5A shows that the field is relatively homogeneous outside the spheres, indicating that the external dipolar field perturbation in an axial plane cutting approximately through the center of a sphere is not as large as the internal field offset. However, inset Fig. 5C shows the variation of the field in a ρ-z plane cutting through the 10-mm-diameter sphere. The dipolar field variation due to the isotropic part of the PGS susceptibility is more evident in this field map, which uses a different scaling to Fig. 5A. From these data, we estimate a value of *χ*_*I*_ = −111 ± 39 ppm.

### Measuring the internal and external field perturbations due to cylindrical PGS shells

**Figure 6:**
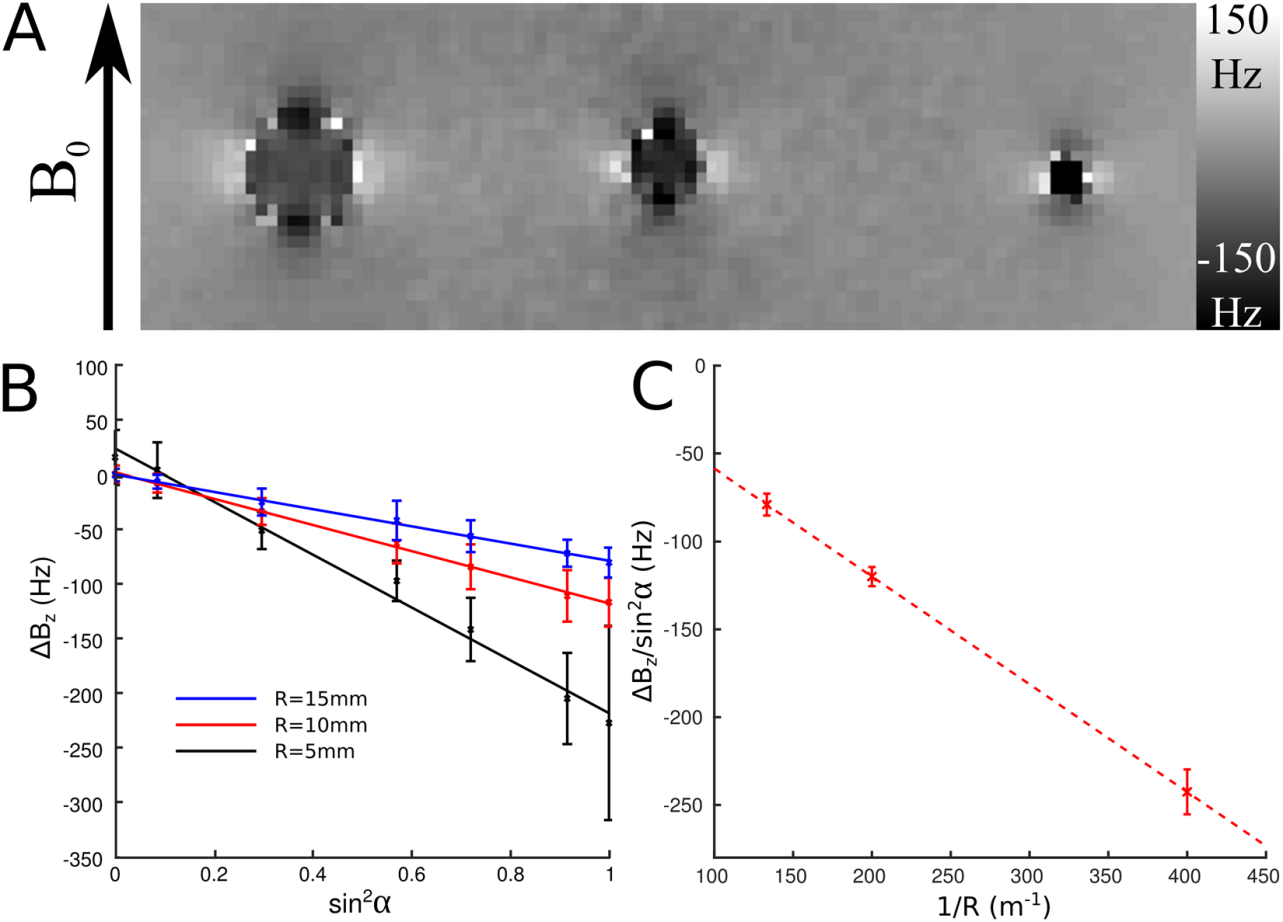
(**A**) Field map showing the field variation (in (Hz) due to glass tubes with radii, *R* (from right to left) of 2.5, 5 and 7.5 mm, covered with a single layer of nominally 25-μm-thick PGS and embedded in a 180 mm diameter agar sphere. The tubes are oriented perpendicular to the *B*_0_-field. (**B**) Variation of the internal field shift 〈Δ*B*_*z*_〉 in each tube as a function of sin^2^ α, where α is the angle between the tube and the *B*_0_-field. Error bars show standard deviation over region inside each tube. C) Variation of the slope of the plot of 〈Δ*B*_*z*_〉 against sin^2^ α (Fig. 2B) with 1/*R*. Error bars show the 68% confidence interval for the fit to each line shown in Fig. 5B.

Figure 6A shows a 46.5 × 149 mm^2^ section of the field map acquired with the three PGS covered tubes oriented perpendicular to *B*_0_. A negative frequency offset, whose magnitude increases with decreasing shell radius is evident in the map, as predicted by Eq. (6). The field perturbation outside the cylinders, which varies as $\frac{\cos \,2\phi }{{\rho }^{2}}$ is also evident in this map. Figure 6B shows plots of the average field offset inside the three different cylinders as a function of sin^2^ α, where α characterizes the orientation of the cylinder with respect to the applied field. These plots show that the frequency offset scales linearly with sin^2^ α with a constant of proportionality which increases in magnitude as *R* decreases in agreement with Eq. (6). Figure 6C shows the gradient of the slopes shown in Fig. 6B as a function of the inverse radius of the cylinders. Linear regression gave a good fit to the data (*R*^2^ > 0.99), confirming the linear relationship and yielded a value of *χ*_*A*_ = −221 ± 11 ppm.

**Figure 7:**
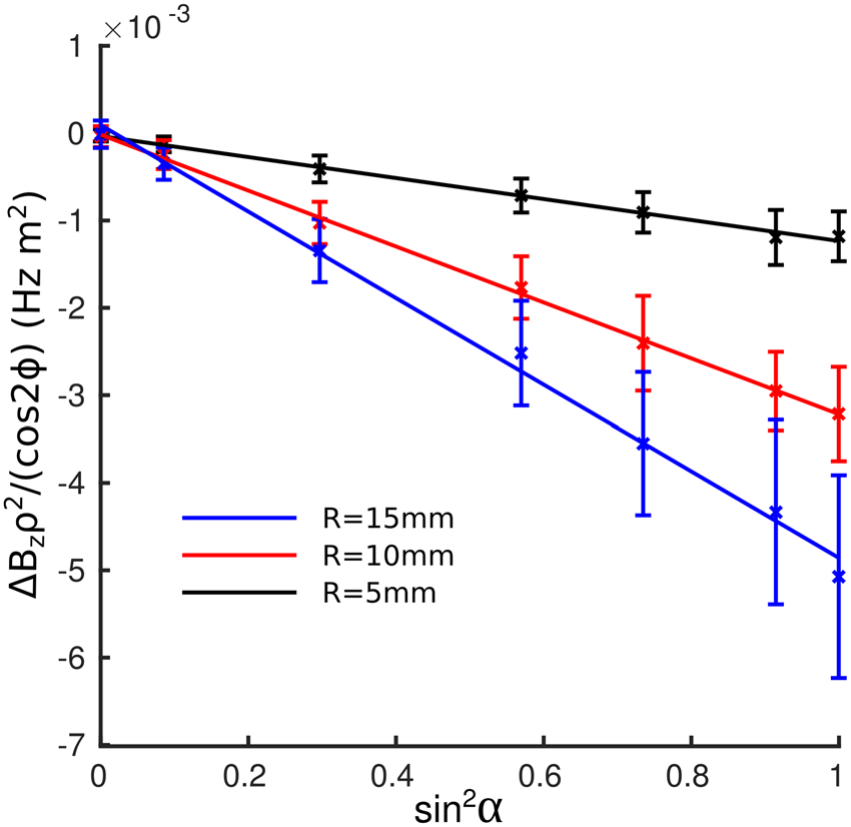
Variation with sin^2^ *α* of the amplitude of the external field perturbation (which varies as cos 2*ϕ*/*ρ*^2^) due to due to glass tubes with radii, *R* of 2.5, 5 and 7.5 mm, covered with a single layer of nominally 25-μm-thick PGS. Error bars show the uncertainty in the fits to the cos 2*ϕ*/*ρ*^2^ variation at each orientation.

Figure 7 shows variation of the amplitude of the fit of the external field variation to cos 2*ϕ*/ρ^2^ with sin^2^ α for the three PGS covered tubes. As expected from Eq. (6) the amplitude varies linearly with ${\sin }^{2}{\rm{\alpha }}$ (R^2^ > 0.99) and increases with the radius of the tube. An F-test showed no evidence of any significant improvement in the model with the addition of a sin^4^ α term which would be expected to occur if an orientation-dependent value for *χ*_*I*_ were used in Eq. (6) (P = 0.9018/0.9998/0.1352 for the 5/10/15 mm cylinders). Based on the slopes of the plots in Fig. 7 and the value of *χ*_*A*_ calculated from the internal frequency offsets, we estimated a value of *χ*_*I*_ = −131 ± 31 ppm.

## Discussion

In this work, we exploited MRI-based field mapping in conjunction with the flexible nature and strong susceptibility anisotropy of pyrolytic graphite sheet to visualize directly the magnetic field perturbations generated by simply-shaped structures composed of anisotropic material when they are exposed to a uniform magnetic field. In all cases the measured field perturbations were in good agreement with analytic expressions that are valid in the regime where the magnitude of the volume susceptibility is much less than unity (which is the case for most paramagnetic and diamagnetic materials at ambient temperatures).

The field perturbation due to an elemental volume of anisotropic material arranged at different angles to the field was experimentally characterized and shown to be different from that produced by purely isotropic material. As shown in Eq. (3) this behavior results from the generation of a magnetization component that is perpendicular to the applied magnetic field, as well as from the orientation dependent modulation of the amplitude of the component of magnetization that is parallel to the field. The perpendicular component of the magnetization is largest in amplitude when the principal component of the cylindrically-symmetric susceptibility tensor is oriented at an angle of 45° or 135° with respect to the magnetic field.

The field perturbations due to spherical and cylindrical shells of anisotropic material in which the principal axis of the susceptibility tensor is oriented normal to the shell surface were also experimentally investigated and shown to display behavior that is very different from that produced by similar structures formed from magnetically isotropic material: the most significant difference being the generation of spatially uniform field offsets inside the shells. The experimental data is consistent with the theoretically predicted field patterns, shown in Fig. 1. In the case of the cylinder, the internal field offset is orientation dependent and largest when the cylinder is perpendicular to the applied field. Even at this orientation, the offset produced by the cylindrical shell is smaller by a factor of 3/4 than that produced by a spherical shell. For both types of structure, the internal frequency offset is proportional to the anisotropic susceptibility, *χ*_*A*_, and to the ratio of the shell thickness to its radius. Detailed analysis (Supplementary Material) shows that the internal frequency offset scales as the logarithm of the ratio of the outer and inner diameters of the shell when the shell’s thickness is not much smaller than its radius. In the case of the spherical shell, the anisotropic susceptibility does not contribute to the external field perturbation, while for the cylindrical shell the anisotropic susceptibility contributes by a factor of 4 less than the isotropic susceptibility to the spatial varying external field. This interesting behavior also results from the generation of a magnetization component that is perpendicular to the magnetic field and whose amplitude varies coherently within the shell. In the case of the cylindrical shell, this produces a component of the magnetization distribution which follows the pattern of variation found in a Halbach cylinder^27^, ${\boldsymbol{M}}={\rm{\Lambda }}(\cos \,\phi \hat{{\boldsymbol{\rho }}}+\,\sin \,\phi \hat{{\boldsymbol{\phi }}})$, with Λ = *H*_0_*χ*_*A*_/2. Similarly, the magnetization distribution in the spherical shell due to the anisotropic part of the susceptibility tensor produces a magnetization distribution varying as ${\boldsymbol{M}}={\rm{\Lambda }}(\cos \,\theta \,\hat{{\bf{r}}}+\frac{1}{2}\,\sin \,\theta \,\hat{{\boldsymbol{\theta }}})$ with Λ = *H*_0_*χ*_*A*_, as is found in a Halbach sphere^27^.

The values of the anisotropic susceptibility, *χ*_*A*_, of the 25-μm-thick PGS measured from the two different forms of spatially varying field found around the slab (−257 ± 21 ppm and −234 ± 18 ppm) were consistent with one another and with those measured from the internal field offset in the spherical (−212 ± 16 ppm) and cylindrical (−221 ± 11 ppm) shells. The *χ*_*A*_-values measured from the sphere and cylinder would be expected to be slight underestimates because of the small gaps which were left between the PGS strips forming the shells to reduce RF screening effects. This meant that there was less PGS material present than was assumed in deriving expressions for the field perturbation based on continuous shells. Using the value of *χ*_*I*_ = −121 ± 10 ppm estimated from the measurement of the dipolar field around the slab and the average of the above *χ*_*A*_-values, yields *χ*_⊥_ = −450 ± 15 ppm and *χ*_||_ = −5 ± 15 ppm. The magnetic susceptibility of commercially supplied PGS has not previously been reported as far as we are aware, but these values are consistent with previous literature on the susceptibility of pyrolytic graphite^25^. The values of *χ*_*I*_ estimated from analysis of the external fields produced by the spherical (111 ± 39 ppm) and cylindrical shells (131 ± 31 ppm) are in general agreement with the value calculated from the measurements on the PGS stack.

The measurements reported here provide some supporting evidence for the models that have previously been used to explain the effects of anisotropic magnetic susceptibility in biological samples. The largest such effects are seen in the white matter of the brain in which myelinated nerves are the source of susceptibility anisotropy. The myelin sheaths of these nerves are formed from lipid bi-layers in which the long lipid chains are radially oriented, such that the principal axis of the susceptibility tensor in the myelin sheath is radially directed and the value of *χ*_*A*_ is negative^6,7^. This is similar to the geometry of the cylindrical PGS shells investigated here, and as a consequence of the larger size and much greater anisotropy of the PGS structures, we were able to directly visualize the uniform negative field offset inside the cylinder (Fig. 6A) and experimentally to confirm the predicted dependence of the field perturbations on the shell geometry and orientation (Figs 6 and 7). This dependence is important since it underpins the interpretation of the time dependence of the gradient echo signal generated from white matter *in vivo*^21,28^. In contrast to the PGS-based model system used here, some NMR signal is generated by the cylindrical shell in myelinated nerves, but the low concentration and short T_2_^*^-relaxation time of the myelin water means that this signal does not contribute significantly to images acquired at standard echo times (>10 ms) in gradient echo imaging. It is consequently the negative intra-axonal frequency that generally makes the dominant contribution to the negative average NMR signal frequency (and related phase effects) seen in white matter regions where the nerve fibers are oriented perpendicular to the applied field^1,7^. This effect also underlies the increased range of signal frequencies and consequent reduction of T_2_^*^ found in these white matter regions compared with those in which the nerve fibers are parallel to the field^29^. More generally, Figs 5A and 6A make it evident that the field variation and associated NMR signal variation produced by microstructural tissue features formed from components of anisotropic magnetic susceptibility will not usually be well characterized by use of a model that represents each image voxel as a homogeneous piece of material of isotropic or anisotropic susceptibility. Since this is the modelling approach that is used in QSM and STI^29^, there are potential problems with accurate characterization of the susceptibility of white matter tracts and adjacent tissues using these techniques^30^, so the results which they produce must be interpreted with care.

The measurements of the field perturbations generated by the PGS slabs make evident the importance of accounting for the effect of the component of the induced magnetization that is perpendicular to the applied field when considering the effect of anisotropic susceptibility – the maximum field perturbation that this component of the magnetization generates at a given radius for fixed *χ*_*A*_ is more than half of the maximum field generated by the parallel magnetization, and the amplitude of field variation due to the perpendicular magnetization is larger than that due to the parallel magnetization for values of ${\rm{\Theta }}$ between ~34 and 71° (and ~109 and 146°). Approaches that analyze the effect of the anisotropic susceptibility in terms of an apparent isotropic susceptibility whose amplitude varies with the orientation of the material with respect to the magnetic field^5,15^ do not therefore properly characterize the field perturbations and may yield erroneous results. For example, applying this approach to a cylindrical shell would yield no internal field offset and a contribution to the cos 2*ϕ*/*ρ*^2^ external field variation whose amplitude varies with sin^4^ α^14^ – no such variation is of course seen experimentally.

The maps which show the uniform field offsets inside spherical shells and the scaling of this offset with the inverse of the shell radius provide direct experimental visualization of the model previously used by Lounila *et al*.^10^ to explain size-dependent spectral shifts in the ^13^C NMR frequency measured from compounds confined within naturally occurring lipoproteins. These shifts result from the anisotropic magnetic susceptibility of the lipid chains that form the lipoprotein shell in such a way that the long axis of the chains, and thus the principal axis of the susceptibility tensor, is always radially oriented in the shell: a geometry that is replicated by the PGS-covered spherical shells. It is worth noting however that the anisotropic susceptibility of lipid membranes is estimated to be around −0.2 ppm^7,10^, which is more than 1000 times less than the value in PGS. The high magnetic anisotropy and relatively low density of PGS means that large field perturbations can be generated with small amounts of material. The work described here highlights new opportunities for improved local passive shimming in MRI^31^, where the additional degrees of freedom provided by the possibility to change the orientation of the induced magnetization could allow better cancellation of field inhomogeneity by appropriate placement of PGS material around the outside of the body. In addition, microscopic PGS shells could potentially be exploited in producing “multi-spectral” contrast agents for MRI. Recent work by Zabow *et al*.^32,33^ has shown how nano-fabricated structures formed from pairs of nickel or iron disks can be used as contrast agents that encode distinguishable spectral signatures. This is possible because of the geometry-sensitive field shift produced between the paramagnetic disks. The capability to produce a geometry-sensitive field shift inside a spherical shell formed from PGS could offer advantages over planar metallic structures, although the RF screening effects of PGS would need to be addressed.

## Electronic supplementary material

Supplementary Information
