# Supplementary material for: Quantifying MRI frequency shifts due to structures with anisotropic magnetic susceptibility using pyrolytic graphite sheet
Source: Sci Rep. 2018 Apr 19;8:6259. doi: 10.1038/s41598-018-24650-2 (PMC5908955; doi:10.1038/s41598-018-24650-2)
Supplement: Supplementary file 1 — Supplementary Information [file 41598_2018_24650_MOESM1_ESM.docx]

**Supplementary Information**

Quantifying MRI frequency shifts due to structures with anisotropic magnetic susceptibility using pyrolytic graphite sheet:

Matthew J. Cronin^1,†^ and Richard Bowtell^1^

^1^ Sir Peter Mansfield Imaging Centre, School of Physics and Astronomy, University Park, Nottingham, NG7 2RD, UK

^†^ Current address: Institute of Imaging Science, Vanderbilt University Medical Center, 1161 21st Avenue South, Medical Center North, AA-1105, Nashville, TN 37232-231, USA

Spherical Shell

For a spherical shell of radius *R* and thickness *t* ( << R) composed of PGS with the axis of strong diamagnetism aligned with the surface normal, the magnetization in the shell is of the form

$\boldsymbol{M}= H_{0}\left( \left( \chi_{A}+\chi_{I} \right)\cos\theta\hat{\mathbf{r}}\boldsymbol{+}\left( \frac{\chi_{A}}{2}-\chi_{I} \right)\sin\theta\hat{\boldsymbol{\theta}} \right)\boldsymbol{.}[S1]$

so that

$$\nabla\boldsymbol{.M=}\frac{{3H}_{0}\chi_{A}\cos\theta}{r} . [S2]$$

Using Eq. [2] the potential generated by this magnetization can be written as:

$$\Phi\left( \boldsymbol{r} \right)= \frac{H_{0}}{4\pi}\left[ \oint_{r^{'}=R_{o}} \frac{\left( \chi_{A}+\chi_{I} \right)\cos\theta'}{\left| \boldsymbol{r}\mathbf{-}\boldsymbol{r}^{\mathbf{'}} \right|}dS^{'}-\oint_{r^{'}=R_{i}} \frac{\left( \chi_{A}+\chi_{I} \right)\cos\theta'}{\left| \boldsymbol{r}\mathbf{-}\boldsymbol{r}^{\mathbf{'}} \right|}dS-\int_{\boldsymbol{V}} \frac{3\chi_{A}\cos\theta'}{r\left| \boldsymbol{r}\mathbf{-}\boldsymbol{r}^{\mathbf{'}} \right|}d^{3}\boldsymbol{r}^{\mathbf{'}} \right], [S3]$$

where $R_{i}=\left( R-t/2 \right)$ is the inner radius of the shell, $R_{o}=\left( R+t/2 \right)$ describes its outer radius and the volume integral is taken over the shell region, where $R_{i}<r<R_{o}$.

Using the Green’s function expansion:

$$\frac{1}{\left| \mathbf{r-}\mathbf{r}^{\mathbf{'}} \right|}=4\pi\sum_{l=0}^{\infty} \sum_{m=-l}^{m=l} \frac{1}{2l+1} \frac{r_{<}^{l}}{r_{>}^{l+1}} Y_{l,m}^{*}(\theta',\phi')Y_{l,m}(\theta,\phi) , [S4]$$

where $Y_{l,m}\left( \theta,\phi\right)$ are Laplace spherical harmonics and $r_{<}=r'$ and $r_{>}=r$ when $r^{'}<r$, and $r_{<}=r$ and $r_{>}=r'$ when $r<r^{'}$, Eq. [S3] can be rewritten as:

$$\Phi\left( \boldsymbol{r} \right)= \frac{H_{0}}{3}\sum_{l=0}^{\infty} \int_{0}^{\pi} 2\pi\cos^{2}\theta' Y_{l,0}^{*}\left( \theta^{'} \right)Y_{l,0}\left( \theta\right)d\theta^{'} \times\left[ \left( \chi_{A}+\chi_{I} \right)\left( \left\{ \frac{R_{o}^{2}r_{<}^{l}}{r_{>}^{l+1}} \right\}_{R_{o},r}-\left\{ \frac{R_{i}^{2}r_{<}^{l}}{r_{>}^{l+1}} \right\}_{R_{i},r} \right)-\chi_{A}\int_{R_{i}}^{R_{o}} \left\{ \frac{3r'r_{<}^{l}}{r_{>}^{l+1}} \right\}_{r,r'}dr' \right]. [S5]$$

The orthogonality of the spherical harmonics means that only the $l=1$ yields a non-zero value of the integral over $\theta^{'}$, and since we are interested in the field inside and outside the shell surface (rather than in the material of the shell) the integral over $r'$ is straightforward to calculate yielding:

$\Phi\left( \boldsymbol{r} \right)= \frac{H_{0}\cos\theta}{3}\times\left[ \begin{matrix} \left( \chi_{A}+\chi_{I} \right)\left( \frac{R_{o}^{3}}{r^{2}}-\frac{R_{i}^{3}}{r^{2}} \right)-\chi_{A}\int_{R_{i}}^{R_{o}} \frac{3{r'}^{2}}{r^{2}}dr' \\ \left( \chi_{A}+\chi_{I} \right)\left( \frac{R_{o}^{2}r}{R_{o}^{2}}-\frac{R_{i}^{2}r}{R_{i}^{2}} \right)-\chi_{A}\int_{R_{i}}^{R_{o}} \frac{3r}{r'}dr' \end{matrix} \right] \begin{matrix} r>R_{o} \\ r<R_{i} \end{matrix}$ [*S*6]

which simplifies to

$\Phi\left( \boldsymbol{r} \right)= H_{0}\cos\theta\times\left[ \begin{matrix} \frac{\chi_{I}}{3}\left( \frac{R_{o}^{3}{-R}_{i}^{3}}{r^{2}} \right) \\ -\chi_{A}r ln\left( \frac{R_{o}}{R_{i}} \right) \end{matrix} \right] \begin{matrix} r>R_{o} \\ r<R_{i} \end{matrix}$ . [*S*7]

The z-component of the field is then given by

$B_{d}=-\mu_{0}\hat{\boldsymbol{z}}.\nabla\Phi\left( \boldsymbol{r} \right)= B_{0}\times\left[ \begin{matrix} \frac{\chi_{I}}{3}\left( \frac{R_{o}^{3}{-R}_{i}^{3}}{r^{3}} \right)\left( 3\cos^{2} \theta- 1 \right) \\ \chi_{A}\ln\left( \frac{R_{o}}{R_{i}} \right) \end{matrix} \right] \begin{matrix} r>R_{o} \\ r<R_{i} \end{matrix}$ . [*S*8]

This leads to Eq. [5], since $R_{o}^{3}{-R}_{i}^{3}\approx3R^{2}t$ and $\ln\left( \frac{R_{o}}{R_{i}} \right)\approx\frac{t}{R}$, when $t\ll R.$

Cylindrical Shell

For a cylindrical shell of radius *R* and thickness *t* ( *<< R*) composed of PGS with the axis of strong diamagnetism aligned with the surface normal and the cylinder oriented at angle $\alpha$ to the magnetic field, the magnetization in the annulus is given by

$$\boldsymbol{M}= H_{0}\left( \left( \chi_{A}+\chi_{I} \right)\sin\alpha\cos\phi\hat{\boldsymbol{\rho}}\boldsymbol{+}\left( \frac{\chi_{A}}{2}-\chi_{I} \right)\sin\alpha\sin\phi\hat{\boldsymbol{\phi}}\boldsymbol{+}\left( \chi_{I}-\frac{\chi_{A}}{2} \right)\cos\alpha\hat{\boldsymbol{z}} \right) , [S9]$$

so that

$\nabla\boldsymbol{.M=}\frac{{3H}_{0}\chi_{A}\sin\alpha\cos\phi}{2\rho} . [S10]$

Following a similar approach to that used in analysis of the spherical shell we find that

$$\Phi\left( \boldsymbol{r} \right)= \frac{H_{0}\sin\alpha}{4\pi}\left[ \oint_{\rho^{'}=R_{o}} \frac{\left( \chi_{a}+\chi_{I} \right)\cos\phi'}{\left| \boldsymbol{r}\mathbf{-}\boldsymbol{r}^{\mathbf{'}} \right|}dS^{'}-\oint_{\rho^{'}=R_{i}} \frac{\left( \chi_{A}+\chi_{I} \right)\cos\phi'}{\left| \boldsymbol{r}\mathbf{-}\boldsymbol{r}^{\mathbf{'}} \right|}dS-\int_{\boldsymbol{V}} \frac{3\chi_{A}\cos\theta'}{2\rho\left| \boldsymbol{r}\mathbf{-}\boldsymbol{r}^{\mathbf{'}} \right|}d^{3}\boldsymbol{r}^{\mathbf{'}} \right], [S11]$$

(where $R_{o}$ and $R_{i}$ are as defined above). Then substituting the Green’s function expansion in cylindrical polar co-ordinates

$$\frac{1}{\left| \mathbf{r-}\mathbf{r}^{\mathbf{'}} \right|}=\frac{1}{\pi} \sum_{m=-\infty}^{\infty} \int_{-\infty}^{\infty} dke^{ik\left( z-z^{'} \right)}e^{im\left( \phi-\phi^{'} \right)}I_{m}(k\rho_{<})K_{m}(k\rho_{>}), [S12]$$

(where $I_{m}$ and $K_{m}$ are the modified Bessel functions and $\rho_{<}=\rho'$ and $\rho_{>}=\rho$ when $\rho^{'}<\rho$, and $\rho_{<}=\rho$ and $\rho_{>}=\rho'$ when $\rho<\rho^{'}$) into Eq. [S11]: and integrating over $z^{'}$ and $\phi^{'}$ (which picks out terms with $m=\pm1$) yields

$$\Phi\left( \boldsymbol{r} \right)= \frac{H_{0}\sin\alpha}{2}\cos\phi\left[ \left[ \left( \chi_{A}+\chi_{I} \right)\left( \left\{ \frac{R_{o}\rho_{<}}{\rho_{>}} \right\}_{R_{o},\rho}-\left\{ \frac{R_{i}\rho_{<}}{\rho_{>}} \right\}_{R_{i},\rho} \right)-\chi_{A}\int_{R_{i}}^{R_{o}} \left\{ \frac{3\rho_{<}}{{2\rho}_{>}} \right\}_{\rho,\rho'}d\rho' \right] \right]. [S13]$$

This simplifies to

$\Phi\left( \boldsymbol{r} \right)= \frac{H_{0}\sin\alpha}{2}\cos\phi\times\left[ \begin{matrix} \left( \chi_{A}+\frac{\chi_{I}}{4} \right)\left( \frac{R_{o}^{2}-R_{i}^{2}}{\rho} \right) \\ -\frac{3\chi_{A}}{2}\ln\left( \frac{R_{o}}{R_{i}} \right) \end{matrix} \right] \begin{matrix} \rho>R_{o} \\ \rho<R_{i} \end{matrix}$ . [*S*14]

The component of the field perturbation that is parallel with $B_{0}$ is given by

$B_{d}=-\mu_{0}\hat{\boldsymbol{Z}}.\nabla\Phi\left( \boldsymbol{r} \right)= B_{0}\sin^{2}\alpha\times\left[ \begin{matrix} \left( \chi_{A}+\frac{\chi_{I}}{4} \right)\left( \frac{R_{o}^{2}-R_{i}^{2}}{2\rho^{2}} \right)\cos2\phi\\ \frac{3\chi_{A}}{4}\ln\left( \frac{R_{o}}{R_{i}} \right) \end{matrix} \right] \begin{matrix} r>R_{o} \\ r<R_{i} \end{matrix}$ , [*S*15]

where $\hat{\boldsymbol{Z}}$ is a unit vector along the applied field direction that is oriented at angle, $\alpha$ with respect to the axis of the cylindrical shell. Equation [*S*15] leads to Eq. [6], since $R_{o}^{2}{-R}_{i}^{2}\approx2Rt$ and $\ln\left( \frac{R_{o}}{R_{i}} \right)\approx\frac{t}{R}$, when $t\ll R.$
